# Evolution of meningococcal carriage in serogroups X and Y before introduction of MenAfriVac in the health district of Kaya, Burkina Faso

**DOI:** 10.1186/s12879-014-0546-8

**Published:** 2014-10-14

**Authors:** Absatou Ky Ba, Idrissa Sanou, Paul A Kristiansen, Lassana Sangaré, Rasmata Ouédraogo, Kalifa Ouattara, Maxime Kienou, Simon Tiendrebeogo, Juliette Tranchot

**Affiliations:** Laboratoire National de Santé Publique, Ouagadougou, Burkina Faso; Centre Hospitalier Universitaire Yalgado, Ouagadougou, Burkina Faso; WHO Collaborating Center for Reference and Research on Meningococci, Norwegian Institute of Public Health, Oslo, Norway; Centre Hospitalier Universitaire Pédiatrique Charles de Gaulle, Ouagadougou, Burkina Faso; Institut de Recherche en Science de la Santé, Ouagadougou, Burkina Faso; Universitaire Polytechnique de BoboDioulasso, Bobo-Dioulasso, Burkina Faso

**Keywords:** Meningococcal meningitis, Serogroups X and Y, Carriage, Risk factor

## Abstract

**Background:**

The objective of this study was to evaluate the carriage of *Neisseria meningitidis* (Nm) serogroups X and Y in the health district of Kaya before the introduction of a serogroup A meningococcal conjugate vaccine in Burkina Faso.

**Methods:**

A repeated cross-sectional meningococcal carriage study was conducted in 2009 in eight randomly selected villages in the health district of Kaya, Burkina Faso. In each of 4 sampling rounds at least 1,500 people were enrolled within a 1-month period.

**Results:**

From a total of 6,686 throat swabs we identified 419 Nm isolates (6.27%). The dominating serogroups were Y (3.19%) and X (1.05%). Overall carriage was higher in the dry season compared with the rainy season (OR, 1.51; 95% CI, 1.06–2.16). Carriage prevalence of serogroups Y and X varied by round and was highest at the end of the dry season (4.92% and 1.22%, respectively). The only risk factor associated with NmX carriage was vaccination status in contrast to serogroup Y, which was associated with age groups 5–9 years and 10–14 years.

**Conclusion:**

The presence of Nm serogroups X and Y, which could replace or be added to the serogroup A, is a warning sign. There is a need to strengthen surveillance and laboratory diagnosis of the various meningococcal serogroups circulating in Africa.

## Background

Meningococcal meningitis is a major public health problem globally [1]. According to the World Health Organization (WHO), the number of cases of meningitis over the last 15 years is estimated to be more than 700,000, with a fatality rate of over 10% and sequelae reported in more than 20% of surviving patients [1]. Overall, about 500,000 cases of meningococcal disease occur each year causing at least 50,000 deaths. Meningococcal meningitis has a case-fatality rate of 5%–10% in industrialized countries, which can reach 20% in the developing world. In addition, 12%–19% of survivors develop long-term neurological sequelae [2],[3]. Sub-Saharan Africa, and in particular the area known as the Lapeyssonnie meningitis belt, is the most affected region. This area, with a population estimated at about 500 million inhabitants, stretches from Senegal to Ethiopia [1],[4]. In countries located in this area, there is an upsurge in cases of meningitis in the dry season every year, which gives a high endemic background [1],[5],[6]. This meningitis is mostly caused by meningococcal serogroup A.

In Burkina Faso, 78,518 cases of meningitis with 8,568 deaths were recorded between 2003 and 2009, a lethality rate of 11%. Nearly 65% of cases were due to *Neisseria meningitidis* serogroup A. The latest epidemic year was 2007, with 26,878 recorded cases and 1,923 deaths. In 2008, 10,401 cases and1,067 deaths were recorded, and there were 4,723 cases and 629 deaths in 2009 (MDSC Meningitis Weekly Bulletin, http://www.who.int/csr/disease/meningococcal). Moreover, Burkina Faso was the first country to experience a major serogroup W outbreak in 2002 [7]-[9], with 13,000 reported cases, of whom 1,400 died (www.who.int/csr/disease/meningococcal/w135). However, 6960 cases with 63% of serogroup W were reported in 2012.

*N. meningitidis* colonizes 8%–25% of healthy individuals, this carriage rate in the oropharynx can be 5%–10% in non-epidemic periods [10]-[12]. In 1998, a carriage study examining nasopharyngeal specimens from 1,818 high school students from hypersporadic counties in the metropolitan area of Atlanta, GA, found the rate of carriage to be 7.7%. Of these, 48% were serogroup Y [13]. However, in 2006–2007, a similar carriage study in high school students found a much lower proportion of serogroup Y carriage [13]. Serogroup X (ST-181) has caused localized outbreaks in certain African countries, including Kenya, Niger, and Ghana but is rarely seen as a cause of disease outside of Africa [14],[15]. A study conducted by Kristiansen et al. in Burkina Faso before the introduction of serogroup A meningococcal conjugate vaccine showed a carriage rate of serogroup Y (2.28%) and that of serogroup X (0.44%) [16]. According to Ministry of Health reports in 2011 and 2012, 11,009 cases of meningitis were notified with 1,388 deaths. No case of *Neisseria meningitidis* serogroup A was observed.

Until 2010 the fight against meningitis was based primarily on halting ongoing epidemics through reactive vaccination with bivalent (AC), trivalent (ACY) and tetravalent (ACYW) polysaccharide vaccines [17].

These vaccines containing capsular polysaccharides have proved safe and effective. However, the immune protection is short and immunity in children is considered sub-optimal. Conjugated vaccines, which bind the polysaccharides to a protein support, induce a stronger immune response in children less than 2 years of age and give a longer protection than polysaccharide vaccines [6],[17],[18]. According to the WHO Regional Office for Africa based in Burkina Faso, 153.5 million people were immunized with the serogroup A meningococcal conjugate vaccine MenAfriVac from 2010 to 2013 in 12 countries (Burkina Faso, Mali, Niger, Nigeria, Cameroon, Chad, Sudan, Ethiopia, Benin, Ghana, Senegal, Gambia). According to the same source, 100 million people should receive the vaccine between 2014 and 2016.

Burkina Faso was the first country to vaccinate the whole population between 1 and 29 years of age with the serogroup A meningococcal conjugate vaccine MenAfriVac [19],[20].

A study of meningococcal carriage was conducted from 2009 to 2011 before and after the introduction of the conjugate vaccine, which showed that serogroup A carriage was eliminated for up to 1 year after mass vaccination, among both vaccinated and non-vaccinated populations [19]. The results published from this study has not yet included sub-analysis of evolution and risk factors for carriage.

The aim of our study was to examine the evolution and risk factors of X and Y meningococcal carriage in the period before vaccination in the health district of Kaya, which is one of the three study sites of the carriage study conducted in Burkina Faso [16]. This site was studied in particular because of higher carriage prevalence of serogroups X and Y compared with the other two study sites, and because these serogroups had not caused any outbreak in the past in Burkina Faso.

## Methods

### Ethics

The study received approval from the Ethics Committee for Health Research in Burkina Faso, the Norwegian Regional Committee for Medical Research Ethics, Southern Norway and the internal Review Board at the Centers for Disease Control and Prevention (CDC) in Atlanta, GA, USA. Informed consent was obtained from all study participants.

### The study site

This is a repeated cross-sectional study with four sampling rounds of 1 month each, which took place from 26 January 2009 to 06 December 2009 in the health district of Kaya, Burkina Faso. The health district of Kaya is located in the northeast of Burkina Faso and the capital of the province is about 100 km from Ouagadougou, the capital of the country. Within the district, eight villages were selected by probability proportional to size. They included: Tamdogo, Terrin Mossi, Forgui, Boulsin, Mastenga, Iryastenga, Foulla and Noungou. In each round, at least 1,500 people meeting the criteria for participation in the study were enrolled.

### Sampling method

In each of the selected villages, all compounds were mapped with the use of a global positioning system (GPS) before the study started. For each round and in each village, 30 compounds were randomly selected using the GPS coordinates.

### Inclusion of participants and administration of questionnaires

The village population was informed about the project through local health workers and community leaders. Each randomly selected compound was visited by study personnel and the purpose of the study was explained. A first questionnaire with general questions about the compound was administered to the head of the compound after his written consent was obtained. The first questionnaire included the following information: number of people living in the household; number of rooms in the household; whether toilets and bathrooms were present; how many people usually slept in the same room; how many people usually slept on the same surface (e.g., bed, mattress); how many people in the household had been affected by meningitis in the last 5 years; and type of fuel used. Then, a second questionnaire was administered for each of the 1- to 29-year-old members of the household, who presented no signs of chronic illness or obvious malnutrition, and lived at the household. Individual written consent or, in the case of children below 18 years of age, the consent of their parent or guardian was first obtained. Each participant was given a paper wristband with a barcode corresponding to a unique identifier number linked to the questionnaire. The second questionnaire queried the following: sex; age; whether respondents had been vaccinated against meningitis in the last 5 years; whether medications had been taken during the last 30 days; whether respondents had been in a place where they talked with several people outside work or school in the past month (e.g., wedding, funeral, market); whether they had made a pilgrimage to the Hajj; occupation (for those 12 years old or older); attendance at school and type of school (for 5- to 19-year-old respondents); and tobacco use (for those 10 years old or older). For children below 18 years the answers were completed by their parent or guardian if necessary.

### Sample collection and analysis

Oropharyngeal samples were obtained by sweeping the posterior pharyngeal wall behind the uvula and one tonsil with a sterile cotton swab (Copan, Italy) as previously described [16]. The swab was immediately plated onto modified Thayer-Martin VCNT agar, containing 3 mg/liter vancomycin, 7.5 mg/liter colistin, 12.5 U/liter nystatin, 5 mg/liter trimethoprim lactate, and Vitox supplement (produced by the WHO Multi-Disease Surveillance Centre, Burkina Faso). In the field, inoculated plates were rapidly incubated in humidified, CO_2_-rich air using 7.0-liter jars (Remel, GA) with a CO_2_-generating system (CO_2_ GEN; Oxoid, UK). Personal digital assistants were used to register the barcode on the participant’s wristband as well as the barcode label used on the inoculated plate. This created a link between the person identifier number and the laboratory specimen number. The jars with the plates were incubated at 37°C in the laboratory within 6 h after sampling. Between 100 and 110 samples were collected daily, 4 days per week, during the 4 weeks of each campaign [16].

### Laboratory analysis

The inoculated agar plates were incubated at 37°C with C0_2_ generators for 24 to 48 hours. Colonies suspected to be Nm were subcultured on blood agar and incubated at 37°C under C0_2_. Suspect colonies on fresh blood agar were tested for oxidase activity, Gram staining, β-galactosidase (ONPG) activity and γ-glutamyltransferase (GGT) activity as described [16]. The serogroup was determined by slide agglutination with capsule-specific antiserum (Remel, GA). Purified isolates were inoculated into two cryovials containing 0.5 – 1 ml Greaves solution [21] and stored at -70°C. After each round, one of the vials was sent to the Norwegian Institute of Public Health (NIPH), Oslo, Norway, on dry ice for confirmation and further analyses. The final laboratory results are based on results from NIPH.

### Statistical analysis

Descriptive analysis of the data was performed; frequency tables for the variables of interest were made for each round. Then, we studied the factors associated with carriage of NmY and NmX using logistic regression with survey methods accounting for the cluster sampling design in STATA v.12.

## Results

### Description of the sample

A total of 6,686 people, of whom 3,730 (55.8%) were females, were included in the study during the 4 rounds. More than half of the participants (55.6%) were less than 10 years old. Of the 5- to 19-year-old participants, 31.28% attended school. Approximately 70% of respondents had been vaccinated with meningococcal polysaccharide vaccine. The characteristics of the sample are given in Table 1.

**Table 1:** **Characteristics of the sample**

Characteristics	Number	Percentage (%)
**Age (years)**		
1–4	1630	24.38
5–9	2088	31.23
10–14	1504	22.49
15 and older	1464	21.90
Total	6686	100
**Schooling**		
Yes	1358	31.28
No	2984	68.72
Total	4342	100
**Vaccination**		
Yes	4579	68.49
No	2032	30.39
Don’t know	75	1.12
Total	6686	100
**Medication**		
Yes	2452	36.67
No	4234	63.33
Total	6686	100

### Meningococcal carriage prevalence

Overall meningococcal carriage in Kaya was 6.27% with a higher prevalence in the dry season (6.03% in round 1 and 8.83% in round 2) compared with the rainy season (5.43% in round 3 and 4.71% in round 4) (odds ratio (OR) dry season: rainy season, 1.51; 95% confidence interval (CI), 1.06–2.16). Overall, carriage of Nm Y was three times higher than that of Nm X. Carriage prevalence varied by round from 0.30% to 1.99% for NmX and from 2.44% to 4.62% for NmY (Table 2). Carriage of other serogroups was lower; 0.92% (range, 0.61–1.35%) for NmA, 0.53% (range, 0.35–0.84%) for NmW and 0.58% (range, 0.30–0.90%) for non-serogroupable Nm.

**Table 2:** **Evolution of the carriage prevalence of** ***Neisseria meningitidis*** **serogroups X and Y in Kaya (Burkina Faso) by rounds**

Round	Number of participants	Nm X	Nm Y
		Number of carriers (%)	Number of carriers (%)
1	1658	5 (0.30%)	49 (2.96%)
2	1710	34 (1.99%)	79 (4.62%)
3	1639	20 (1.22%)	40 (2.44%)
4	1679	11 (0.66%)	45 (2.68%)
Total	6686	70 (1.05%)	213 (3.19%)

### Risk factors for carriage

The only risk factor associated with NmX carriage was vaccination status (Table 3). Participants who reported having received a meningococcal polysaccharide vaccine in the past 5 years had higher NmX carriage prevalence than the non-vaccinated population (P = 0.046). No association with gender, medications or level of education was found. However, the carriage of Nm Y appears to have a link with age unlike the X Nm (Tables 3 and 4).

**Table 3:** **Risk factors for the carriage of** ***Neisseria meningitidis*** **serogroup X**

Variables	Numbers	Positive %	OR (IC.95)	Value of P
**Sex**				
Female	3730	0.97	1	
Male	2956	1.15	1.19 [0.78–1.83]	0.359
**Age**				
1–4	1630	1.04	1	
5–9	2088	1.15	0.74 [0.56–2.16]	0.739
10–14	1504	1.06	0.93 [0.60–1.73]	0.931
15 and older	1464	0.89	0.63 [0.39–1.84]	0.634
**Medication**				
Yes	2452	1.20	1	
No	4234	0.77	1.56 [0.81–3.03]	0.156
**Schooling**				
Yes	1358	0.81	1	
No	2984	1.11	1.37 [0.34–5.53]	0.611
**Vaccination**				
No	2032	0.64	1	
Yes	4579	1.24	1.96 [1.01–3.78]	0.046

**Table 4:** **Risk factors for the carriage of** ***Neisseria meningitidis*** serogroup Y

Variables	Numbers	Positive %	OR [95% CI]	Value of P
**Sex**				
Female	3730	2.92	1	
Male	2956	3.52	1.21 [0.98–1.50]	0.070
**Age**				
1–4	1630	1.78	1	
5–9	2088	3.64	2.09 [1.39–3.14]	0.004
10–14	1504	3.79	2.17 [1.03–4.59]	0.043
15 and older	1464	3.48	1.99 [1.26–3.13]	0.009
**Medication**				
Yes	2452	2.94	1	
No	4234	3.33	1.14 [0.78–1.67]	0.445
**Schooling**				
Yes	1358	3.17	1	
No	2984	3.95	1.26 [0.92–1.73]	0.128
**Vaccination**				
No	2032	3.10	1	
Yes	4579	3.25	1.05 [0.74–1.49]	0.747

## Discussion

Our study, which was conducted over a period of about 1 year concluded that the overall carriage rate of NmY was three times higher than that of NmX.

In our study the age groups of 5–9 years and 10–14 years accounted for 31.2% and 22.5% of the participants, which indicates a wide participation of these age groups in a rural site like Kaya. Indeed, when they are not attending school, the children are at home with no particular occupation. We also found that in these age groups, children cooperated more often during the oropharyngeal swabbing compared with those younger than 5 years.

In this study we noticed an increase of the serogroup X carriage from 0.30% in January–February 2009 (round 1) to 1.99% in April–May 2009 (round 2). This period, which corresponds to the dry season, is the period during which peaks of meningococcal disease are usually recorded [10]. NmX carriage then declined but was still high during August–September 2009 (round 3) in the rainy season, the period during which the meningitis disease incidence is generally low. The climate may be directly linked to increased carriage and epidemic meningococcal meningitis, but also indirectly linked by influencing communities to settle down [10],[22]. The increase in NmX carriage may be explained by the proximity of the health district of Kaya to Niger, which experienced a large outbreak of serogroup X meningitis in 2006 [23]. In fact, the health district of Kaya experienced an outbreak of serogroup X meningitis during the 2010–2011 epidemic season.

The low carriage prevalence of non-serogroupable Nm reflects the differences in epidemiology of carriage between and within countries. In Europe one would expect 50% of the carriage isolates to be non-groupable. Within Burkina Faso, the proportion of non-groupable Nm was significantly higher in urban Bogodogo compared to rural sites and most of non-groupable isolates were assigned to sequence types known to lack the gene coding for capsule synthesis [16].

The identification of vaccination status as a risk factor for NmX carriage is disputable because this finding is dependent on correct reporting (recall bias) and because we did not control vaccination cards. Furthermore, meningococcal polysaccharide vaccines have previously shown a questionable impact on pharyngeal carriage, and serogroup X has not yet been included in any licensed vaccine. In Burkina Faso, polysaccharide vaccines are only used for mass vaccination in outbreak situations and the Ministry of Health confirmed that mass vaccination had been conducted in Kaya in the past 5 years. Although vaccinated people would not be able to acquire immunity against NmX, background immunity in both vaccinated and non-vaccinated people would be the same so we cannot argue that vaccination is a clinically significant risk factor for NmX carriage.

The only risk factor associated with NmY carriage prevalence was age; we found a statistically significant difference between age groups. Carriage prevalence was highest in the age group of 10–14 years followed by the 5–9 year-old age group. Our study is comparable to that conducted in Canada in 2003 [24] as well as the one carried out in Mali in 1970 that found a statistical link between carrying serogroup A meningococci and the age group of 5–9 years [25]. In addition the results of a study in Colombia between 1994 and 2006 showed that over 50% of cases of meningococcal serogroup Y were young people under 20 [26]. Serogroup Y was the dominant carriage serogroup in the district of Kaya and this might explain why we found a significant variation in carriage by age for serogroup Y and not for X [16].

In our study the use of medicines did not affect X or Y meningococcal carriage. People gathering at schools, markets, and public places promotes airborne transmission of meningococci, but curiously, attending school was not associated with higher X or Y meningococcal carriage.

## Conclusion

*Neisseria meningitidis* serogroups X and Y circulated among healthy carriers in the district of Kaya during a 1-year period before the introduction of a serogroup A conjugate vaccine. Carriage prevalence of both serogroups increased during the 2009 epidemic season. Round 2, held from April to May 2009 showed high carriage rates for the two serogroups. Because countries neighboring Burkina Faso such as Niger and Ghana have recently experienced the circulation and epidemic outbreak of serogroup X meningitis [23],[27], the presence in our study of NmX is alarming as new serogroups with epidemic potential could replace or be added to serogroup A. This underlines the need to strengthen surveillance and laboratory diagnosis in Africa to detect all serogroups of meningococci. This preliminary study should promote further evaluation of the role of vaccination on the carriage of meningococci. A decrease in the carriage through vaccination with a conjugate vaccine could help reduce outbreaks of epidemics of meningitis in the meningitis belt.

## Authors’ contribution

AB analyzed the data and drafted the manuscript. PAK, IS, RO, and LS participated in the design of the study. AB IS, RO, LS were responsible for collecting the carriage isolate. PAK was responsible for coordination of the carriage study. AB contributed with training and supervision. ST and PAK participated in the statistical analysis. KO and MK contributed to the laboratory analysis. JT contributed to the amendment of the manuscript. All authors helped revise the manuscript and approved the final version.
